# De-icing performance evolution with increasing hydrophobicity by regulating surface topography

**DOI:** 10.1080/14686996.2024.2334199

**Published:** 2024-04-02

**Authors:** Wei Weng, Xiaoyang Zheng, Mizuki Tenjimbayashi, Ikumu Watanabe, Masanobu Naito

**Affiliations:** a Research Center for Macromolecules and Biomaterials, National Institute for Materials Science (NIMS), Tsukuba, Japan; b Center for Basic Research on Materials, National Institute for Materials Science (NIMS), Tsukuba, Japan; c Research Center for Materials Nanoarchitectonics (MANA), National Institute for Materials Science (NIMS), Tsukuba, Japan

**Keywords:** Superhydrophobic, de-icing, surface topography, interfacial strength distribution, ice adhesion, ice detachment simulation

## Abstract

It is of great significance to grasp the role of surface topography in de-icing, which however remains unclear yet. Herein, four textured surfaces are developed by regulating surface topography while keeping surface chemistry and material constituents same. Specifically, nano-textures are maintained and micro-textures are gradually enlarged. The resultant ice adhesion strength is proportional to a topography parameter, i.e. areal fraction of the micro-textures, owing to the localized bonding strengthening, which is verified by ice detachment simulation using finite element method. Moreover, the decisive topography parameter is demonstrated to be determined by the interfacial strength distribution between ice and test surface. Such parameters vary from paper to paper due to different interfacial strength distributions corresponding to respective situations. Furthermore, since hydrophobic and de-icing performance may rely on different topography parameters, there is no certain relationship between hydrophobicity and de-icing.

## IMPACT STATEMENT

The role of surface topography in de-icing is verified to be determined by the interfacial strength distribution between ice and surface experimentally and numerically, unveiling the relationship between hydrophobicity and de-icing.

## GRAPHICAL ABSTRACT

**Figure:**
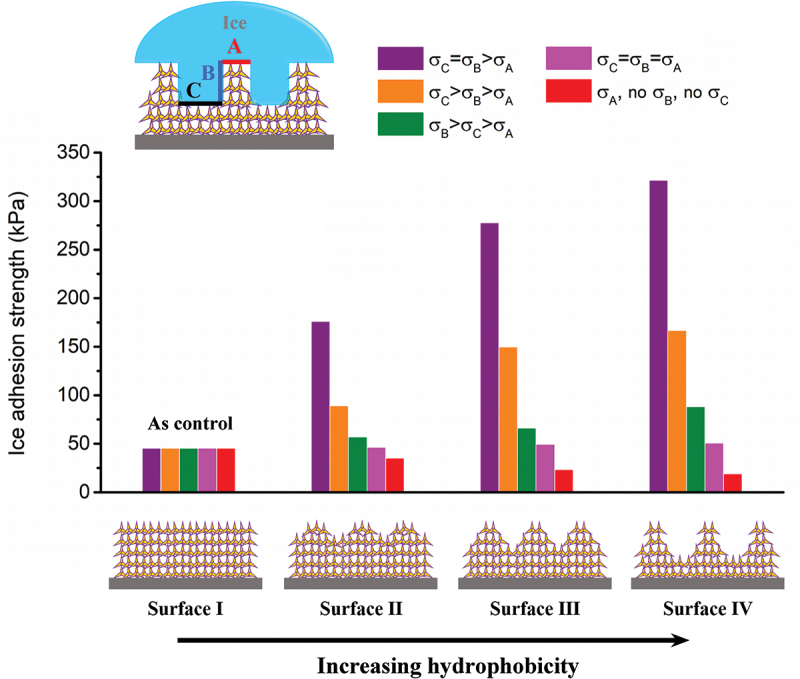

## 1. Introduction

Superhydrophobicity has long been correlated with icephobicity that includes anti-icing and de-icing [1,2]. On one hand, superhydrophobic (SHPO) surfaces have presented promising anti-icing properties, e.g. lowering ice nucleation temperature and prolonging the freezing delay time of sessile water droplets, repelling impacting subcooled droplets, and jumping-removal of condensed droplets via coalescence [3–5], though these may deteriorate under high humidity [6,7]. On the other hand, SHPO surfaces’ de-icing performance should be taken into consideration when icing occurs, which is ubiquitous at subzero temperatures via solidification, desublimation, or solid precipitation [8]. Many claimed that much lowered ice adhesion strength (IAS) was attained due to the realization of superhydrophobicity [9–21]. On the contrary, others found that hydrophobic or even few hydrophilic surfaces outperformed SHPO surfaces in de-icing [22–31]. The controversy emerged at the very beginning and continues hitherto.

Over 50 IAS data for SHPO surfaces from references in the last decade are listed in Figure S1 and Table S1, among which more than 80% lie between 20 kPa and 350 kPa no matter what kind of SHPO surfaces were designed and what kind of test conditions were applied. To be mentioned, IAS < 100 kPa is regarded as low ice adhesion [32], and self-removal of ice by vibration or wind requires IAS < 20 kPa [33]. Generally, IAS of rigid and high-surface-energy materials such as aluminum, copper, and steel exceeds 1000 kPa [33,34]. Apparently, when compared to them, SHPO surfaces possess much better de-icing performance. However, it is plausible to use smooth hydrophobic surfaces as controls because SHPO surfaces are typically achieved by introducing surface textures into smooth hydrophobic surfaces, which represents two indispensable elements of SHPO surfaces: surface chemistry of low surface energy and surface topography of nano-textures, micro-textures, or nano-/micro-textures [35]. The IAS of hydrophobic surfaces using low-surface-energy polymers, such as silicones is between 30 and 100 kPa and can be hundreds of kPa for fluorinated hydrophobic surfaces [1,36]. Therefore, a large majority of the IAS of SHPO surfaces and hydrophobic counterparts overlap, raising complexity to unveil if superhydrophobicity benefits de-icing.

In light of this, it is vital to grasp the roles of surface chemistry and surface topography in de-icing. For a smooth surface, the maximum water contact angle (CA) reported in the literature is ⁓130º [37,38]. The effect of surface chemistry on IAS has been well investigated by Robert E. Cohen and co-workers. It was found that IAS correlated strongly with the work of adhesion required to remove a liquid water droplet from the test surface, i.e. with the quantity (1 + cos*θ*_*rec*_) [39]. *θ*_*rec*_ is the receding angle. Since increasing CA does not always increase *θ*_*rec*_, increasing hydrophobicity from the aspect of surface chemistry does not necessarily lower IAS.

For a textured surface, CA can be enlarged to 150º, a threshold for superhydrophobicity, and even > 170º [40,41]. The effect of surface topography on IAS has been studied using pillared structures due to their high tunability. Kripa K. Varanasi and co-workers adjusted the pillar size and spacing, and the resultant IAS had a strong linear trend with the total surface area [42]. Moreover, Hyuneui Lim and co-workers regulated pillars’ top diameter, and the IAS was proportional to the areal fraction of top base [43]. Although their decisive surface topography parameters were different, both works showed that increasing hydrophobicity caused decreasing IAS. In addition, much effort has been devoted to pursue the low-IAS surfaces by altering topography and material simultaneously [16,44,45], where the relationship between surface topography and IAS could not be straightforwardly established. Therefore, more study is needed to reveal the role of surface topography in de-icing and the underlying mechanism.

Herein, four non-pillared textured surfaces are developed with the same materials but different surface topographies. Among them, nano-textures are maintained but micro-textures are gradually enlarged resulting in the evolving surface topography and the increasing hydrophobicity accordingly, which facilitates direct links not only between surface topography and de-icing but also between hydrophobicity and de-icing. Experimental results show that the decisive topography parameter for the four textured surfaces is the areal fraction of micro-textures, and by increasing it IAS is linearly increased. Moreover, assisted by ice detachment simulation, this decisive topography parameter is verified to be determined by the interfacial strength distribution between ice and test surface, which accounts for the varied decisive topography parameters from paper to paper.

## 2. Materials and methods

### 2.1. Preparation of textured surfaces

Four textured surfaces were all composed of ZnO tetrapods (pana-tetra WZ-0501, AMTEC Co., Ltd.) and polydimethylsiloxane (PDMS, HC2100, Toray Industries, Inc.) using the techniques we reported previously [46]. Briefly, suspensions of ZnO and PDMS in ethyl acetate solvent were first prepared. Then, dip-coating and spin-coating methods were applied to make coatings on glass slides (S7224, Matsunami Glass Ind., Ltd.). Finally, samples were dried before use. Three parameters, i.e. concentration of suspensions, coating speed, and coating repeat time, were controlled to adjust surface topography. In practice, coating speed including the rotation speed of spin-coating and the withdrawal speed of dip-coating could not be large. Otherwise, the uniformity of surfaces would be poor. As for setting repeat time, the priority was to guarantee the same thickness of the four textured surfaces. The most flexible parameter was the concentration of suspensions. With increasing concentration, micro-textures were enlarged after an initial decrease. Only a medium value leaded to nano-texture-only surface. Hence, not only surface topography but also uniformity and thickness were considered, resulting in the final preparation techniques.

### 2.2. Preparation of FAS-modified glass surface

First, 1 H, 1 H, 2 H, 2 H-perfluorodecyltriethoxysilane (FAS, Sigma-Aldrich) was mixed with anhydrous ethanol, resulting in a 2 wt.% FAS solution. Then, glass slides were immersed into the FAS solution for 24 h at room temperature. Afterward, the treated glass slides were taken out of the solution, washed with ethanol, and finally dried at 120°C for 2 h. Thus, FAS-modified glass slides were obtained.

### 2.3. Preparation of PDMS surface

A coating solution was made by dissolving 4.5 g PDMS in 24 mL ethyl acetate. The solution was spin-coated on glass slides with a rotation speed of 200 rpm, a coating time of 300 s and a repeat number of 4. At last, the samples were dried at 80°C for 2 h before use. To be mentioned, the PDMS coating had a thickness of 20 μm, which was similar to that of textured surfaces.

### 2.4. Morphology and wettability characterization

Tabletop scanning electron microscope (SEM, Miniscope TM3000, Hitachi, Japan) and field emission scanning electron microscope (FESEM, JSM-7001F, JEOL, Japan) were used to observe the morphology of ZnO tetrapods and all surface samples. Laser confocal scanning microscope (Optelics HYBRID C3, Lasertec, Japan) was applied to obtain three-dimensional (3D) surface topography of textured surface samples. The morphology of textured surfaces after de-icing test was also tested by the laser confocal scanning microscope. Moreover, roughness parameters including *Ra* (arithmetic average of profile height deviations from the mean line) and *Sa* (arithmetic average of height difference of each point from the mean plane) were measured by the built-in LMeye7 software.

Static contact angle (CA), sliding angle (SA), advancing angle (*θ*_*adv*_) and receding angle (*θ*_*rec*_) were measured using a contact angle meter (DMs-401, Kyowa Interface Science, Japan), where water droplets (deionized water, 18 MΩ ∙ cm at 25 ºC) of 8 μL were used, and room temperature of 22 ± 1°C and relative humidity of 52 ± 2% were kept. Each CA, SA, *θ*_*adv*_, or *θ*_*rec*_ test was repeated at least five times. Dynamic photos of water droplets (8 μL) sliding off test surfaces were captured by a high-speed camera (Mini AX, FASTCAM, Photron, Japan). Moreover, water droplets on test surfaces with decreasing temperature were monitored. The surface temperature was decreased from 20°C to −10°C at 2°C min^−1^ by a Peltier stage (ZTC-1212, Z-MAX, Japan), while the ambient temperature and relative humidity were respectively fixed at 22 ± 1°C and 52 ± 2%. The real-time CA change was tested by the contact angle meter and the real-time droplet image was shot by the high-speed camera that was positioned horizontally. Also, water penetration into textured surfaces was studied. Deionized water with 0.05 wt.% of Rhodamine B was used. One situation was that water droplets (10 μL) were dropped on textured surfaces at 22 ± 1°C and 52 ± 2% relative humidity and then slid off the surfaces by tilting. The other situation was as follows: first, water droplets (10 μL) were dropped on textured surfaces in the same ambient condition. Second, the surface temperature was lowered to −10°C at 2°C min^−1^, after which the temperature was returned to room temperature naturally. Third, the droplets were removed by tilting. The distribution of Rhodamine B remaining in the textured surfaces was detected by laser confocal fluorescence microscope (TCS SP5, Leica, Germany).

### 2.5. De-icing performance test

First, frost plus bulk water ice was formed and tested. Tests were carried out in an experimental stall, in which ambient temperature and relative humidity were maintained at 22 ± 1°C and 52 ± 2%, respectively. Cuvettes were put on the test surface samples mounted on a Peltier stage. Meanwhile, water of 1 mL was added. Afterward, cooling was started and the sample temperature was decreased to −10°C at 2°C min^−1^. And −10°C was kept for 4 h to guarantee the water solidification. Then, digital force gauge (DST-50N or DST-200N, IMADA Co., Ltd., Japan) was used to detach the cuvette-encased ice columns (1 cm × 1 cm × 1 cm) with a horizontal probe impact speed of 0.5 mm s^−1^. Finally, the recorded peak force divided by ice area (1 cm × 1 cm) was taken as the IAS data. Second, another ice type, bulk water ice, was made in an environmental chamber (FX420N, ETAC, Japan) to see if the role of surface topography in de-icing changes with changing ice type. In detail, cuvettes were vertically placed on the test surface samples in the chamber at −25°C for 30 min. After that, water of 1 mL was added into the cuvettes and let the icing process last for 4 h, during which the chamber temperature was kept at −25 ± 0.3°C and the chamber relative humidity was at 60 ± 2.5%. Same ice detachment handling was applied. Deionized water (18 MΩ ∙ cm at 25 ºC) was used in de-icing tests. And, every IAS test was repeated at least seven times.

## 3. Results and discussion

### 3.1. Surfaces with increasing hydrophobicity

**Figure 1.**
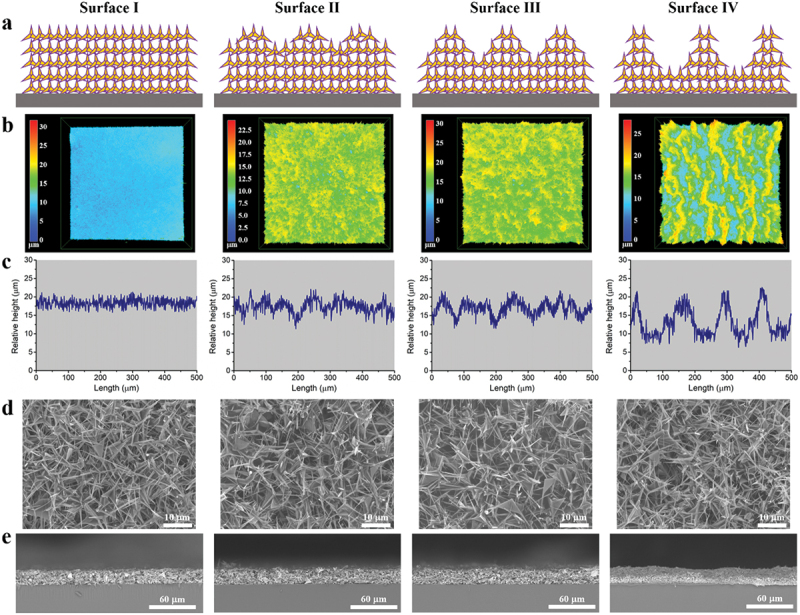
(a) Schematics of four textured surfaces composed of PDMS-coated ZnO tetrapods. (b) 3D images of four textured surfaces by laser scanning confocal microscopy. The test areas are 750 μm × 750 μm. (c) Surface height profiles of four textured surfaces. (d) FESEM images of four textured surfaces. (e) Cross-sectional SEM images of four textured surfaces.

As it is well known, enlarging roughness factor (ratio of actual surface area to projected area) brings about the increasing CA if surfaces are hydrophobic [35]. Moreover, water droplets atop surfaces initially being in Wenzel state jump to Cassie state when the roughness factor surpasses a threshold [35], after which the CA is determined by the areal fraction of air underneath the droplet. With increasing the liquid-air areal fraction, the CA multiplies [38]. Commonly, enlarging roughness factor and increasing liquid-air areal fraction are contradictory. In our previous work [46], we facily solved it by creating both wider and deeper micro-pores stepwise (Figure 1(a)), resulting in the synchronous growth of roughness factor and liquid-air areal fraction. Thus, monotonically increasing hydrophobicity was achieved, which is for the first time among non-pillared surfaces.

Two materials, ZnO tetrapods and PDMS, were used with a fixed weight ratio of 9:1 for all textured surfaces, wherein ZnO tetrapods were wrapped by PDMS. ZnO tetrapods are characterized by its four-spine structure, and a typical view is shown in Figure S2. The length of spines could be several to dozens of micrometers. For simplification, surfaces are deemed to be constructed by PDMS-coated ZnO tetrapods as sketched in Figure 1(a), where the orange three-pointed stars represent ZnO tetrapods and the purple outlines correspond to PDMS coating. The difference among these surfaces is the micro-pores formed on the top. 3D images using a laser confocal scanning microscope are shown in Figure 1(b). The test areas were 750 μm × 750 μm for all samples. Since height is visualized by colors, it is discernible that surface I is the least rough surface, and the rest are getting rougher from surface II to III and to Ⅳ. The corresponding height profiles were drawn in Figure 1(c), from which it can be learnt that surface I has negligible micro-pores but the others have manifest micro-pores. Specifically, the diameters of micro-pores in surfaces II, III, and Ⅳ are 40.7 ± 12.4, 53.1 ± 14.0 and 97.0 ± 12.8 μm, respectively (Figure S3). And the respective depths of micro-pores are 1.6 ± 0.9, 3.5 ± 1.1 and 7.0 ± 0.6 μm.

FESEM images of four textured surfaces are shown in Figure 1(d), where stacked PDMS-coated ZnO tetrapods are obvious. And no big difference can be drawn among their high-magnification morphology, which is opposite to their distinctive low-magnification morphology (Figure 1(b)). Furthermore, since the radii of ZnO tetrapods’ spines (Figure S2) and the voids among stacked ZnO tetrapods due to geometric hindrance are basically less than 1 μm (Figure S4), surface I is regarded as nano-textured. As for surfaces II, III, and Ⅳ, they share nano-textures with surface I and further have micro-pores as micro-textures, making them nano-/micro-textured. Additionally, four textured surfaces have the same thicknesses of 20 μm (Figure 1(e)) to exclude the effect of thickness on de-icing properties. The roughest topography of surface Ⅳ can be verified by the magnified cross-sectional SEM images (Figure S5) as well.

**Figure 2.**
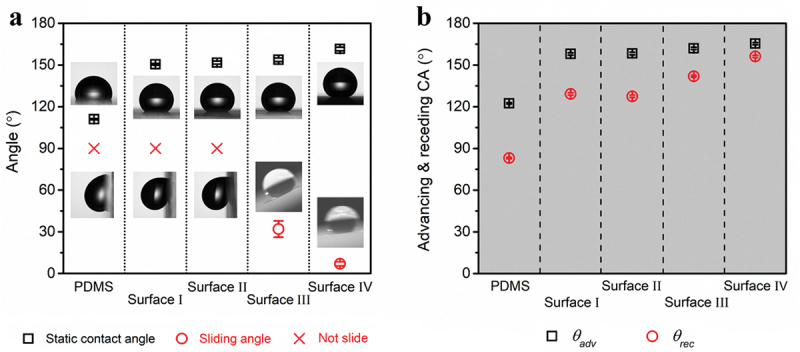
(a) Static CAs and SAs of PDMS surface and four textured surfaces. The insets are test images using water droplets of 8 μL. (b) Advancing angles (*θ*_*adv*_) and receding angles (*θ*_*rec*_) of PDMS surface and four textured surfaces.

CAs and SAs were tested and are shown in Figure 2(a). PDMS surface as smooth hydrophobic surface was prepared and served as a control sample, which is shown in Figure S6 and has a thickness of 20 μm. CAs are 111.0º, 150.0º, 151.7º, 153.8º, and 161.0º for PDMS surface, surfaces I, II, III, and Ⅳ, respectively. Since the CAs become larger and the SAs turn smaller, increasing hydrophobicity for the four textured surfaces is demonstrated. In addition, data of *θ*_*adv*_ and *θ*_*rec*_ are listed in Figure 2(b), from which surfaces I and II exhibit large contact angle hysteresis (CAH), and surface Ⅳ has the smallest one in accordance with the SA test result.

### 3.2. De-icing properties

**Figure 3.**
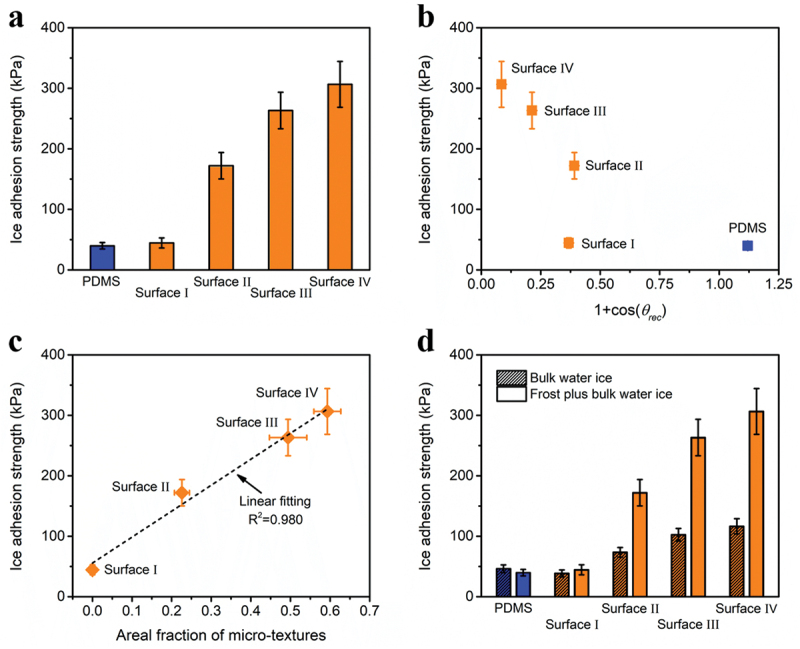
(a) IAS data for PDMS surface and four textured surfaces. (b) Experimental dots of IAS vs. (1 + cos*θ*_*rec*_) for PDMS surface and four textured surfaces. (c) Experimental dots and fitting line of IAS vs. areal fraction of micro-textures for four textured surfaces. (d) Comparison of IAS data between frost plus bulk water ice and bulk water ice.

De-icing test setup is schematically shown in Figure S7. Cuvette-encased ice columns of 1 cm × 1 cm × 1 cm were formed on test surfaces, which were mounted on a Peltier cooler kept at −10°C. Afterward, the peak force needed to dislodge ice columns was recorded and divided by ice cross-sectional area. Thus, IAS was obtained. The resulting IAS data are shown in Figure 3(a), which are 39.8 ± 5.3, 44.5 ± 8.3, 172.0 ± 21.9, 263.3 ± 30.1, and 306.5 ± 37.9 kPa for PDMS surface, surfaces I, II, III, and Ⅳ, respectively. Apparently, deteriorating de-icing performance is obtained as hydrophobicity increases. Moreover, smooth hydrophobic surface, i.e. PDMS surface, exhibits smaller IAS than surface I (the least rough one) mainly due to its softness [33]. To confirm this, glass slides were FAS-modified, turning them from hydrophilic to hydrophobic. The resultant FAS-glass surface has CA of 107.3 º and *θ*_*rec*_ of 78.4º, which are comparable to PDMS surface. However, the IAS of FAS-glass surface is 91.4 ± 13.2 kPa that is much higher than that of PDMS surface.

Previous works have shown that IAS is proportional to the work of adhesion for smooth surfaces [33,39]. Hence, dots of IAS vs. (1 + cos*θ*_*rec*_) were drawn in Figure 3(b). Notably, surfaces I, II, III, and Ⅳ do not abide by this, which is consistent with other reports employing textured surfaces [14,16,44]. Since the four textured surfaces are all composed of PDMS-coated ZnO tetrapods and have same ZnO to PDMS ratios, the difference among their IAS data should be ascribed to their different surface topographies. Plots of IAS vs. roughness factor for the four textured surfaces are shown in Figure S8, and IAS vs. *Ra* was tried in Figure S9. No direct rules were noticed for both cases. In contrast, as seen from Figure 3(c), the correlation between IAS and areal fraction of micro-textures is manifestly linear. The micro-textures, i.e. micro-pores, are illustrated in Figure 1(a), and their areal fractions can be calculated based on wettability and topography data. The deduction details can be found in Section S1 of the Supporting Information and Figure S10. They are 0.226 ± 0.019, 0.494 ± 0.047, and 0.593 ± 0.034 for surfaces II, III, and Ⅳ, respectively.

It has proved that the formation of ice strongly affects IAS [47]. Several types of ice have been used in ice adhesion test, including bulk water ice [48,49], impact ice [49,50], frost plus impact ice [51,52] and frost plus bulk water ice [42,44]. In the above IAS tests frost plus bulk water ice was used. Here, IAS data of bulk water ice was supplemented (Figure 3(d)) to see if the role of surface topography in de-icing changes with changing ice type. There are two main differences between the formation of two ice types. First, the icing temperature was −25°C for bulk water ice, which was −10°C for frost plus bulk water ice. Second, the ambient condition during icing was −25°C and 60% relative humidity for bulk water ice, which was 22°C and 52% relative humidity for frost plus bulk water. Such high ambient temperature was meant to produce frost.

The IAS data using bulk water ice are 46.2 ± 6.4, 38.5 ± 5.7, 73.5 ± 8.0, 102.5 ± 10.3 and 116.4 ± 12.6 kPa for PDMS surface, surfaces I, II, III, and Ⅳ, respectively. For PDMS surface, bulk water ice exhibits a bit higher IAS than frost plus bulk water ice mainly due to the lower icing temperature. The effect of icing temperature on ice adhesion has been largely investigated, and generally the ice adhesion increases with decreasing icing temperature [48,50]. However, the reason is complex, which may include the temperature dependence of surface forces, stress concentration, thickness of QLL (quasi-liquid water layer) and defect density at the interface [48]. As on any type of textured surfaces, frost plus bulk water ice always possesses higher IAS than bulk water ice. This is consistent with the fact that in a humid atmosphere the icing of textured surfaces leads to very large IAS values [38,44]. Frost was formed inside the textures, succeeded by the interaction between frost and bulk water ice, resulting in an interlocking effect, which surpasses the temperature influence. Moreover, the IAS growth rates are 16%, 134%, 157%, and 163% for surfaces I, II, III, and Ⅳ, respectively. Apparently, nano-textured surface has the least deterioration because it has the best resistance against water/condensation intrusion [44,53]. As seen from Figure S11, the IAS of bulk water ice on the four textured surfaces also has a linear relationship with the areal fraction of micro-textures. Therefore, though different types of ice were applied, the role of surface topography in ice adhesion is unchanged.

### 3.3. Experimental study on surface topography’s role

**Figure 4.**
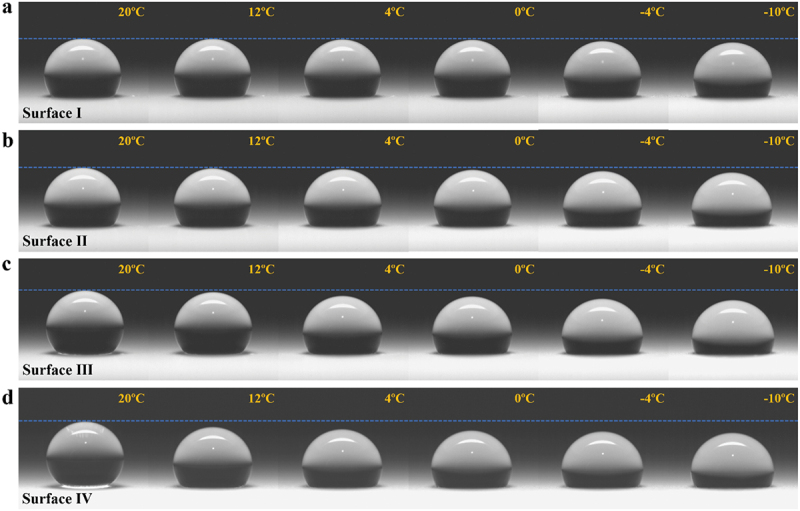
Evolution of water droplets (10 μL) with decreasing temperature from 20°C to − 10°C on (a) Surface I, (b) Surface II, (c) Surface III and (d) Surface Ⅳ.

To probe the effect of surface topography, it is needed to observe water-surface interaction. As seen from Figure 4, water droplets’ evolution with decreasing temperature on the four textured surfaces was monitored. Here, water droplets of 10 μL and cooling rate of 2°C min^−1^ were used. And the ambient condition was similar to that for the IAS test of frost plus bulk water ice. Apparently, both CA and volume of water droplets underwent a change because the height and contact width changed with decreasing temperature. And these changes are moderate for surfaces I and II, but considerable for surfaces III and Ⅳ.

**Figure 5.**
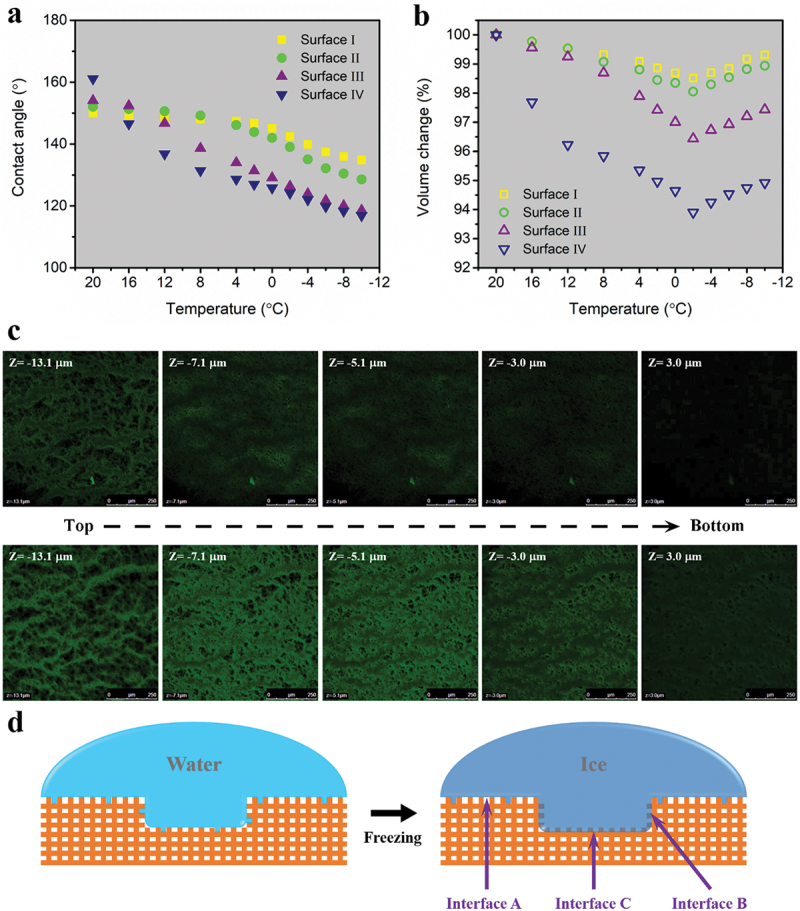
(a) CA change and (b) Volume change with decreasing temperature for four textured surfaces. (c) 3D distribution of fluorescent dye residue in surface Ⅳ after removing water droplets at room temperature (the upper part) and undergoing a cooling-recovery cycle (the lower part). (d) Schemes showing that water intrudes into nano-textures to a small extent but fully occupies micro-textures during cooling, and three interfaces are formed between ice and test surface after water freezing.

Specifically, the CAs and volumes varying with temperature are shown in Figure 5(a,b), respectively. The former was directly measured by CA meter and the latter was calculated when the droplets were deemed as spherical caps [54]. With respect to CA, surface I is the most reluctant one to change and surface Ⅳ has the largest decrease. A turning point at 4°C can be found, before which surface I experienced a little CA drop. However, for surface Ⅳ a large portion of CA reduction happened before 4°C, giving a hint that the air pockets in nano-textures are more stable than in micro-textures [44,55]. As for volume, water droplets dwindled gradually until −2°C even droplets were absorbing water vapor from environment under the test condition. Considering the room temperature of 22°C and relative humidity of 52%, condensation on test surfaces occurred when the surface temperature was lower than 11.7°C (dew point). Therefore, it is believed that water penetrated into the textured surfaces during cooling enabled by condensation-based impalement mechanism in a supersaturation condition, which began at higher temperatures than equilibrium due to premature condensation [56–58]. Furthermore, surface I has the least water impalement and then surface II, surface III and surface Ⅳ in a sequence of getting more severe, which is in line with micro-textures’ morphology.

Additionally, the water penetration was investigated via laser confocal fluorescence microscope by checking the distribution of fluorescent dye residue in the textured surfaces after removing water droplets mixed with a slight amount of Rhodamine B. At room temperature, water droplets only sit upon the asperities of surface Ⅳ as the fluorescence intensity descreases fast with the probe depth (the upper part of Figure 5(c)). However, the intensity has a significant increase before a gradual decrease shown in the lower part of Figure 5(c), where surface Ⅳ experienced a cooling-recovery cycle. Thereby, water impalement is evidently verified during cooling.

As illustrated in Figure 5(d), nano-textures have little water intrusion and micro-textures are fully occupied by water. Subsequently, when water turns into ice, there are three interfaces, A, B and C, between test surface and ice. To dislodge ice columns, it needs to destroy them all. Since the tested IAS corresponds to the peak force, what matters is the interface that needs the largest force to detach ice from it. Conceivably, the bonding at interfaces B and C is much stronger than at interface A because of the explosive vaporization upon recalescence and the volumetric expansion during crystallization [59,60]. Also, interface C needs larger force to decouple than interface B due to its larger area. As a result, interface C determines the IAS and its areal fraction becomes a decisive parameter.

### 3.4. Numerical study on surface topography’s role

As abovementioned, there are three interfaces, A, B and C, between ice and test surface (Figure 5(d)). Accordingly, there are three interfacial strengths, *σ*_*A*_, *σ*_*B*_ and *σ*_*C*_. Since no ice fracture was observed, the ice shedding was fulfilled by adhesive failure. To narrow down the possibilities, it is sensible to assume *σ*_*A*_ is the smallest among the three interfacial strengths. Therefore, three cases need to be considered, i.e. *σ*_*C *_= *σ*_*B*_>*σ*_*A*_, *σ*_*C*_>*σ*_*B*_>*σ*_*A*_ and *σ*_*B*_>*σ*_*C*_>*σ*_*A*_. The adopted simulation model is shown in Figure S12, and the processing of ice detachment simulation can be found in Section S2 of the Supporting Information.

**Figure 6.**
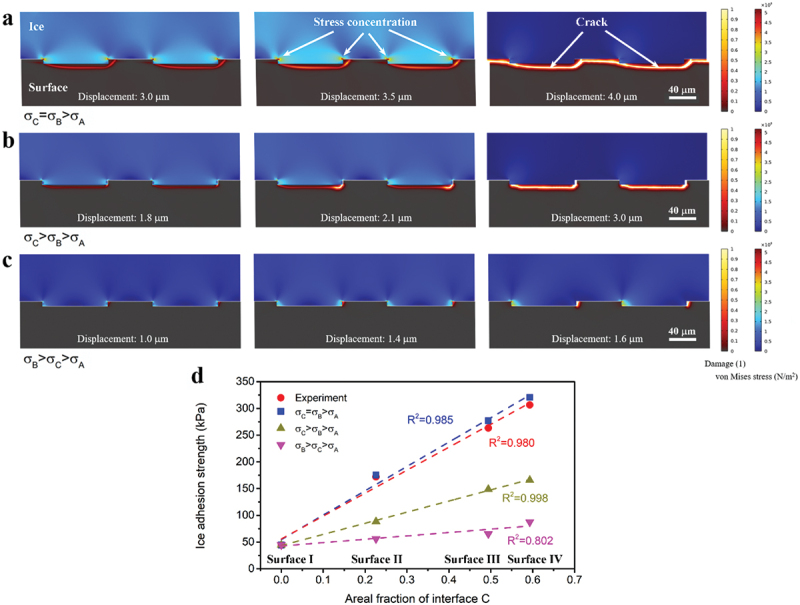
Simulated ice detachment processes corresponding to (a) case of *σ*_*C*_=*σ*_*B*_>*σ*_*A*_, (b) case of *σ*_*C*_>*σ*_*B*_>*σ*_*A*_ and (c) case of *σ*_*B*_>*σ*_*C*_>*σ*_*A*_. Here topography of surface Ⅳ was applied. The color bar of damage corresponds to the splitting process of the textured surfaces. The lighter the color the bigger the damage. The color bar of von Mises stress corresponds to the stress distribution of the ice layer. Large stress concentration is shown at the bulged edges of ice-surface interface. (d) Comparison between simulated and experimental IAS data. Linear fitting of IAS vs. areal fraction of interface C was tried for all cases.

Figure 6(a–c) illustrate the ice detachment processes corresponding to the cases of *σ*_*C*_=*σ*_*B*_>*σ*_*A*_, *σ*_*C*_>*σ*_*B*_>*σ*_*A*_ and *σ*_*B*_>*σ*_*C*_>*σ*_*A*_, respectively. Here, topography of surface Ⅳ was applied. In Figure 6(a), at a displacement of 3.0 μm, interface A has been detached from the ice, and interfaces B and C begin to split, which is represented by the red lines. At a displacement of 3.5 μm, crack grows and propagates along interfaces B and C. And at a displacement of 4.0 μm, crack spreads over the whole surface, resulting in the full ice detachment from the surface. In Figure 6(b), *σ*_*C*_ controls the ice detachement process because *σ*_*C*_ is the largest one. In the same way, *σ*_*B*_ dominates in Figure 6(c). Furthermore, simulation was also conducted on topographies of surfaces I, II, and III, and the IAS data were computed accordingly (Figure 6(d)). Clearly, the case of *σ*_*C*_=*σ*_*B*_>*σ*_*A*_ well matches the experiment. It is worth noting that the case of *σ*_*C*_>*σ*_*B*_>*σ*_*A*_ produces a more linear relationship between IAS and areal fraction of interface C, which however deviates from the real situation by underestimating the contribution of interface B. Thereby, bonding strengthening is demonstrated in the micro-textures (both interfaces B and C) that are vulnerable to water intrusion.

**Figure 7.**
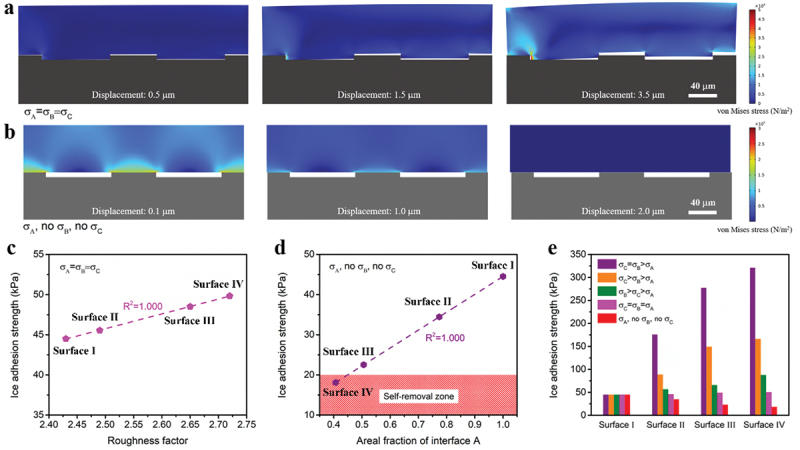
Simulated ice detachment processes corresponding to (a) case of *σ*_*C*_=*σ*_*B*_=*σ*_*A*_ and (b) case of only *σ*_*A*_. Here topography of surface Ⅳ was applied. (c) Simulated IAS data for the case of *σ*_*C*_=*σ*_*B*_=*σ*_*A*_ and linear fitting between IAS and roughness factor. (d) Simulated IAS data for the case of only *σ*_*A*_ and linear fitting between IAS and areal fraction of interface A. (e) Simulated IAS data for all five cases.

Additionally, situations of *σ*_*C*_=*σ*_*B*_=*σ*_*A*_ and only *σ*_*A*_ (*σ*_*C*_=*σ*_*B*_ = 0) were investigated as shown in Figure 7 using the same simulation model. In Figure 7(a), crack emerges at interfaces A, B, and C and grows with increasing displacement until the ice is fully detached from the surface. As for Figure 7(b), the ice detachment is achieved by merely overcoming *σ*_*A*_. Moreover, the computed IAS data for the case of *σ*_*C*_=*σ*_*B*_=*σ*_*A*_ is proportional to the roughness factor (Figure 7(c)), in other words, the total surface area. And the simulated IAS data for the case of only *σ*_*A*_ increases with the areal fraction of interface A linearly (Figure 7(d)).

It is noticeable that different orderings of *σ*_*A*_, *σ*_*B*_, and *σ*_*C*_ result in different decisive surface topography parameters. If *σ*_*C*_=*σ*_*B*_>*σ*_*A*_, which is the example of this work, the decisive parameter is the areal fraction of interface C. If *σ*_*C*_=*σ*_*B*_=*σ*_*A*_, which is found to be the example of [42], the decisive one is the total surface area. And if the situation is only *σ*_*A*_, which resembles the example in [43], the decisive parameter is the areal fraction of interface A. Therefore, the role of surface topography in de-icing is determined by the interfacial strength distribution. Furthermore, the relationship between hydrophobicity and de-icing can be learnt. In the case of *σ*_*C*_=*σ*_*B*_>*σ*_*A*_ of this work, with increasing the areal fraction of interface C, IAS increases and hydrophobicity enhances, leading to a deviation between the two performances. Regarding the case of *σ*_*C*_=*σ*_*B*_=*σ*_*A*_ [42] and the case of only *σ*_*A*_ [43], with increasing respective decisive parameters, IAS increases but hydrophobicity decreases, which means the two performances share the same trend. Consequently, increasing hydrophobicity from the aspect of surface topography does not necessarily lower IAS.

At last, all simulated IAS data for five cases, i.e. *σ*_*C *_= *σ*_*B*_>*σ*_*A*_, *σ*_*C*_>*σ*_*B*_>*σ*_*A*_, *σ*_*B*_>*σ*_*C*_>*σ*_*A*_, *σ*_*C *_= *σ*_*B *_= *σ*_*A*_, and only *σ*_*A*_ (*σ*_*C *_=*σ*_*B*_ = 0), are listed in Figure 7(e). Briefly, the minimum *σ* value in the simulation was fixed. So did the maximum *σ* value if any. And the middle one was always one half of the maximum value, if any. Accordingly, IAS data of surface I are the same in all cases, which serves as a control. On the contrary, IAS data of surfaces II, III, and Ⅳ vary a lot when changing interfacial strength distribution. Looking at surface Ⅳ, in case of *σ*_*C*_=*σ*_*B*_>*σ*_*A*_, its IAS is 320.6 kPa, which turns to 49.8 kPa and 18.1 kPa corresponding to the cases of *σ*_*C*_=*σ*_*B*_=*σ*_*A*_ and only *σ*_*A*_, respectively. Though the practical interface between ice and test surface is more complex, the possibilities listed herein have revealed that different interfacial strength distributions result in largely varied de-icing performance.

### 3.5. Influence of surface robustness

**Figure 8.**
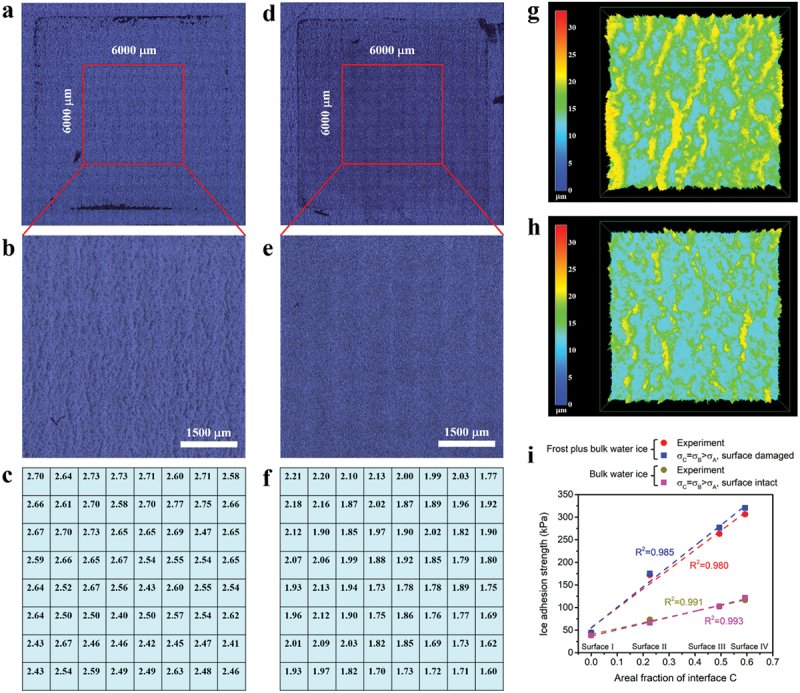
(a) The whole test zone, (b) The central part of the test zone and (c) *Sa* distribution over the central part of the test zone of surface Ⅳ after de-icing test using bulk water ice. (d) The whole test zone, (e) the central part of the test zone and (f) *Sa* distribution over the central part of the test zone of surface Ⅳ after de-icing test using frost plus bulk water ice. Each *Sa* value corresponds to an area of 750 μm × 750 μm. 3D morphology of surface Ⅳ after de-icing tests using (g) Bulk water ice and (h) Frost plus bulk water ice. (i) Plots of experimental and simulated IAS vs. areal fraction of interface C and linear fitting was tried for all cases.

Morphology of surface Ⅳ after de-icing test was probed by laser confocal scanning microscope, which is shown in Figure 8(a,d) corresponding to bulk water ice and frost plus bulk water ice, respectively. The outlines of ice column-occupied zones are obvious for both situations. After removing ice columns, trenches were observed on the surface when dealing with bulk water ice as seen from a magnified image of the central part of the test zone (Figure 8(b)). Contrarily, the surface became less rough after dislodging frost plus bulk water ice (Figure 8(e)). Moreover, *Sa* was tested and each test area was 750 μm × 750 μm. Consequently, 64 tests in total were conducted for each sample (Figure 8(c,f)). The *Sa* of the as-prepared surface Ⅳ is 2.64 ± 0.20 μm. Therefore, the surface was almost intact after de-icing test using bulk water ice, which however underwent a damage by frost plus bulk water ice. 3D images taken after de-icing test were also obtained (Figure 8(g,h)) and they are consistent with the other evidence.

In this regard, the possibility of surface damage should be considered in the ice detachment simulation, which actually has been introduced in Figure 6(a–c) simulating the detachment of frost plus bulk water ice. Regarding *σ*_*C*_=*σ*_*B*_>*σ*_*A*_ (Figure 6(a)), the surface underneath interfaces C and B was set to be damaged because *σ*_*C*_ and *σ*_*B*_ were so strong that the surface damage happened instead. Thereby, the surface underneath interfaces C and B split represented by the red lines. And for *σ*_*C*_>*σ*_*B*_>*σ*_*A*_ (Figure 6(b)), the surface underneath interface C was handled to be destructed. In Figure 6(c) of *σ*_*B*_>*σ*_*C*_>*σ*_*A*_, it was the surface underneath interface B. When the interfacial strength was small, the surface underneath such interface survived and only a clean separation was left. Considering frost formed in the textures, the interface between ice and surface can go deep into the textures, producing a new interface, at which the detachment happens practically. Therefore, the surface damage relates to the interlocking effect in the presence of condensation/frost.

For frost plus bulk water ice, the experimental IAS data are proportional to the areal fraction of micro-textures, which is ascribed to the localized bonding strengthening with an expression of *σ*_*C*_=*σ*_*B*_>*σ*_*A*_ verified by simulation. As for bulk water ice, the experimental IAS data also present a linear increase with the areal fraction of micro-textures (Figure S11). Same surface properties were input into the simulation while *σ*_*C*_, *σ*_*B*_ and *σ*_*A*_ were lowered to meet the experimental data, which also guaranteed the surface was free of damage. The computed IAS data are shown in Figure 8(i). Again, the experiment meets the case of *σ*_*C*_=*σ*_*B*_>*σ*_*A*_. As a result, the role of surface topography is unchanged whether the surface will be damaged or not because the former is about the interfacial strength distribution and the latter is about a comparison between interfacial strength and surface robustness.

## 4. Conclusions

To answer if superhydrophobicity benefits de-icing, the role of surface topography in de-icing is thoroughly investigated both experimentally and numerically. Four textured surfaces using same materials are developed with increasing hydrophobicity by gradually enlarging micro-textures while keeping nano-textures. Here are what we got: 1) The role of surface topography represented by a decisive surface topography parameter is demonstrated to be determined by the interfacial strength distribution between ice and test surface. The decisive parameter varies from paper to paper, which can be, for example, areal fraction of the convex part of a surface, areal fraction of the concave part of a surface, or total surface area. 2) There is no certain relationship between hydrophobicity and de-icing. On one hand, the two performances may rely on different topography parameters. On the other hand, even though they depend on a same topography parameter, the two performances can go in same or opposite directions. 3) Altering interfacial strength distribution is able to cause largely varied de-icing performance. 4) To reach self-removal of ice, reducing apparent interfacial strength, diminishing ice-surface contact and avoiding localized strengthening are promising routines.

## Supplementary Material

Supplemental Material
