# Supplementary material for: De-icing performance evolution with increasing hydrophobicity by regulating surface topography
Source: Sci Technol Adv Mater. 2024 Apr 2;25(1):2334199. doi: 10.1080/14686996.2024.2334199 (PMC10989202; doi:10.1080/14686996.2024.2334199)
Supplement: Supplemental Material [file TSTA_A_2334199_SM6954.docx]

Supporting Information

De-icing performance evolution with increasing hydrophobicity by regulating surface topography

Wei Weng^1,*^, Xiaoyang Zheng^2^, Mizuki Tenjimbayashi^3^, Ikumu Watanabe^2^, Masanobu Naito^1, *^

^1^Research Center for Macromolecules and Biomaterials, National Institute for Materials Science (NIMS), 1-2-1 Sengen, Tsukuba, Ibaraki, 305-0047, Japan

^2^Center for Basic Research on Materials, National Institute for Materials Science (NIMS), 1-2-1 Sengen, Tsukuba, Ibaraki, 305-0047, Japan

^3^Research Center for Materials Nanoarchitectonics (MANA), National Institute for Materials Science (NIMS), 1-1 Namiki, Tsukuba, Ibaraki, 305-0044, Japan

Corresponding authors: weng.wei@nims.go.jp, naito.masanobu@nims.go.jp

Table of Contents

**This PDF file includes:**

Supporting Information Text

Table S1

Figures S1 to S12

**Section S1.** Calculation of areal fraction of micro-pores for surfaces Ⅱ, Ⅲ and Ⅳ

The Cassie-Baxter equation for a composite surface with two components is as follows:

$cos\theta^{*}=\emptyset_{1}\cos\theta_{1}+\emptyset_{2}\cos\theta_{2}$, (1)

where *θ^*^* is the apparent contact angle of the composite surface, *φ_1_* and *φ_2_* are the areal fractions of component 1 and component 2, respectively, *θ_1_* and *θ_2_* are the contact angles of component 1 and component 2, respectively. Also, *φ_1_* + *φ_2_* = 1. For surface Ⅳ, micro-texture was assigned as component 1 and nano-texture as component 2 as illustrated in Figure S10. Since water droplets on surface Ⅳ was in Cassie state, *θ_1_* was 180º. And *θ^*^* was tested to be 161º. Assuming the nano-texture of surface Ⅳ is similar to the nano-texture of surface Ⅰ, *θ_2_* is 150º. Then, we have

$\cos\left( {161}^{^{\circ}} \right)=\emptyset_{1}\cos\left( 180^{^{\circ}} \right)+\left( 1-\emptyset_{1} \right)\cos\left( {150}^{^{\circ}} \right)$. (2)

As a result, *φ_1_* of 0.593 was derived. For surfaces Ⅱ and Ⅲ, we deduced *φ_1_* from their surface topography parameters since neither Wenzel’s equation nor Cassie-Baxter equation can solve it. Specifically, roughness factor, the ratio of actual surface area to its projected area, was used. A simplified scheme is shown in Figure S10. Figure S10a represents a nano-textured surface resembling surface Ⅰ, and Figure S10b illustrates a nano-/micro-textured surface that is like surfaces Ⅱ, Ⅲ and Ⅳ. And their nano-textures are same. The roughness factor of surface Ⅰ was measured to be 2.43, and be 2.72 for surface Ⅳ. Also, they have a relationship:

$2.72=2.43+2h\emptyset_{1}X/r$, (3)

where *h* is the depth of micro-pores, *r* is the radius of micro-pores, and *X* denotes the micro-pore wall’s contribution to roughness factor per unit. Here, micro-pores were supposed to have cylinder shape. For surface Ⅳ, *h* is 7.0 μm, *r* is 48.5 μm, and *φ_1_* is 0.593, then *X* equals 1.69. Subsequently, *φ_1_* of surface Ⅱ was calculated by replacing 2.72 with 2.49 (the roughness factor of surface Ⅱ), and using *h* of 1.6 μm and *r* of 20.35 μm, resulting in *φ_1_* of 0.226. Furthermore, *φ_1_* of surface Ⅲ was calculated by replacing 2.72 with 2.65 (the roughness factor of surface Ⅲ), and using *h* of 3.5 μm and *r* of 26.55 μm, resulting in *φ_1_* of 0.494. Moreover, standard errors were evaluated. Finally, 0.226 ± 0.019, 0.494 ± 0.047 and 0.593 ± 0.034 were obtained for areal fractions of micro-pores in surfaces Ⅱ, Ⅲ and Ⅳ, respectively.

**Section S2.** Ice detachment simulation

Model for ice detachment simulation is shown in Figure S12, containing geometry and boundary conditions. The ice detachment process was simulated using a finite element software package (COMSOL Multiphysics, version 6.0). Resistive forces were considered during the ice detachment process including adhesive force between surface and ice, and fracture force of surface. With respect to adhesive force, a traction-separation law was used. This traction-separation law was implemented via a contact pair at the interface, including an adhesion node describing how robust the adhesive force was and a decohesion node describing the force required to de-bond the interface. During the detachment process, the traction force increased linearly with a stiffness 𝐾_𝑃_ until reaching the critical decohesion force. Thereafter, the opening crack continuously expanded from the failure initiation displacement 𝑢_0_, and the traction force decreased as a function of the damage parameter 𝑑. The interface failed when the stiffness decreased to zero at the ultimate displacement 𝑢_𝑓_.

The displacement 𝑢 consists of tensile (𝑢_𝑡_) and shear (𝑢_𝑠_) relative displacement at the interface:

$u=\sqrt{u_{t}^{2}+u_{s}^{2}}$. (4)

The mixity of tensile (𝑢_𝑡_) and shear (𝑢_𝑠_) relative displacement is defined as:

$\alpha=u_{s}/u_{t}$. (5)

The failure initiation displacement can be calculated as:

$u_{0}=u_{t0}u_{s0}\sqrt{\frac{1+\alpha^{2}}{u_{s0}^{2}+{(\alpha u_{t0})}^{2}}}$, (6)

where 𝑢_𝑡0_ = σ_t_/𝐾_𝑃_, and 𝑢_s0_ = σ_s_/𝐾_𝑃_. σ_t_ and σ_s_ are tensile and shear stengths, respectively. The ultimate displacement 𝑢_𝑓_ is computed with Benzeggaph-Kenane criterion:

$u_{f}=\frac{2}{K_{P}u_{0}}[G_{ct}+\left( G_{cs}-G_{ct} \right)\left( \frac{\alpha^{2}}{1+\alpha^{2}} \right)]$, (7)

where G_ct_ and G_cs_ are tensile and shear energy release rates, respectively. The stiffness at the interface depends on the displacement history that is captured by the maximum displacement 𝑢_𝑚𝑎𝑥_ at each point on the interface. Thus, the damage parameter 𝑑 can be defined as:

$d=\frac{u_{f}(u_{max}-u_{0})}{u_{max}(u_{f}-u_{0})}$. (8)

Therefore, the stiffness of the interface for the shear component becomes

$K_{s}=\left\{ \begin{aligned} K_{P}\Longleftarrow u_{max}\leq u_{0} \\ {(1-d)K}_{P}\Longleftarrow u_{0}<u_{max}<u_{f} \\ 0\Longleftarrow u_{max}>u_{f} \end{aligned} \right.$. (9)

And for the tensile component, it is

$K_{t}=\left\{ \begin{aligned} K_{s}\Longleftarrow u_{t}>0 \\ K_{P}\Longleftarrow u_{t}\leq0 \end{aligned} \right.$. (10)

Brittle fracture of surface was considered using a damage model. The model regularized the crack propagating by a phase field approximation, which enabled the crack path independent of the mesh elements, making the simulation efficient and stable. The damage model was described by the critical energy release rate (𝐺_𝑐_) and the tensile strength that was determined by the damage evolution function and the evolution of the phase field.

Here are data used for the simulation. For ice, Young’s modulus is 8.72 GPa and Poisson’s ratio is 0.31. For surface made of PDMS/ZnO, Young’s modulus is 9.31 GPa, Poisson’s ratio is 0.4, tensile strength $\sigma_{t}$ is 1.0 MPa, and shear strength $\sigma_{s}$ is 0.6 MPa. As to the weakest interface between ice and surface, tensile energy release rate $G_{ct}$ is 1 J/m^2^, and shear energy release rate $G_{cs}$ is 1 J/m^2^. As for the strongest interface between ice and surface if it exists, tensile energy release rate $G_{ct}$ is 10 J/m^2^, and shear energy release rate $G_{cs}$ is 10 J/m^2^. And for the medium interface between ice and surface if it exists, tensile energy release rate $G_{ct}$ is 5 J/m^2^, and shear energy release rate $G_{cs}$ is 5 J/m^2^. Critical energy release rate $G_{c}$ for surface is 11.77 J/m^2^. The mixity $\alpha$ is 2.284. The above parameter input relates to the ice detachment of frost plus bulk water ice. When dealing with bulk water ice, tensile energy release rate $G_{ct}$ and shear energy release rate $G_{cs}$ are 0.8 J/m^2^ for the weakest interface between ice and surface. And tensile energy release rate $G_{ct}$ and shear energy release rate $G_{cs}$ are 7.5 J/m^2^ for the strongest interface between ice and surface if it exists. The rest are the same. Simulation model is a 2D model with plane strain 2D approximation. All of the model geometries were meshed using approximately 2.0×10^5^ second-order triangular solid elements. As for model dimensions, the depth and diameter of micro-textures were directly taken as the pit height and pit diameter (Figure S12), respectively. And the spacing was calculated based on the areal fraction of micro-textures.

**Table S1.** IAS data of superhydrophobic (SHPO) surfaces from the literature.

| Details of SHPO surfaces | Water CA | IAS |
| --- | --- | --- |
| Composite of CeO_2_ nanoparticles and perfluoroalkyl methacrylic copolymer (Zonyl 8740) [1] | 152.4º | ~ 80 kPa |
| Chemically etched Al plate with fluorination treatment [1] | 153.2º | ~ 50 kPa |
| Composite of Ag nanoparticles and perfluoroalkyl methacrylic copolymer (Zonyl 8740) [1] | 155.1º | ~ 90 kPa |
| Composite of TiO_2_ nanoparticles and silicone rubber [1] | 154.8º | ~ 110 kPa |
| Sub-micron grain-like polytetrafluoroethylene film [2] | 152.8º | 770 kPa |
| Etched 6061 Al plate with fluorination treatment [3] | 152.1º | ~ 35 kPa |
| ZrO_2_ nano-powder incorporated fluoropolymer [3] | 153.2º | ~ 80 kPa |
| Micro-grooved Si surface with fluorination treatment [4] | 154.0º | 807 kPa |
| SiO_2_/polyhedral oligomeric silsesquioxane composite coating [5] | 160º | 74 kPa |
| Etched 1060 Al plate with fluorination treatment [6] | 164.4º | 220 kPa |
| Carbon black/TiO_2_/CeO_2_ nanoparticle-incorporated silicone rubber [7] | ~150º | 48 kPa |
| Octadecyltrichlorosilane film [8] | 155º | 42.5 kPa |
| Polymerized hexamethyldisiloxane coating on etched Al [9] | 158º | 100 kPa |
| Micro/nano pillars on Si wafer [10] | ~150º | 495 kPa |
| Composite of silicone rubber and SiO_2_ nanoparticles [11] | 158º | 243 kPa |
| Composite of silicone rubber and Al_2_O_3_ nanoparticles [12] | 156º | 65.5 kPa |
| Etched Al plate with fluorination treatment [13] | 164º | 146 kPa |
| Etched Al plate with palmitic acid treatment [13] | 161º | 314 kPa |
| Zn-coated steel plate with silicone functionalization [14] | 155º | 98 kPa |
| Nanoparticle-covered poly(polydimethylsiloxane  methacrylate) film [15] | ⁓155º* | 50 kPa |
| Anodized Al plate with fluorination treatment [16] | 156º | 40 kPa |
| Square pillars on Cu plate [17] | 156º | 946 kPa |
| Carbon nanotube-covered stainless steel mesh [17] | 155º | 173 kPa |
| Etched Al plate with silicone rubber coating [18] | 165º | 102 kPa |
| Nanoflake-structured Al plate [19] | 169º | 105.5 kPa |
| Nanoflake-coated microarray-patterned Al plate [19] | 173º | 75 kPa |
| Perfluoropolyether-treated pillars (ϕ, 90 nm) on quartz [20] | 151º | 100 kPa |
| Perfluoropolyether-treated pillars (ϕ, 50 nm) on quartz [20] | 160º | 30 kPa |
| Perfluoropolyether-treated pillars (ϕ, 30 nm) on quartz [20] | 169º | 10 kPa |
| Microblock structured Al sheet [21] | 156º | 228.3 kPa |
| Nanohair structured Al sheet [21] | 158º | 127.3 kPa |
| Microblock and nanohair structured Al sheet [21] | 164º | 35.7 kPa |
| Fluorinated SiO_2_ nanoparticles [22] | ~ 162º | 150 kPa |
| Fluorinated SiO_2_/polydimethylsiloxane coating [23] | 155.3º | 26.3 kPa |
| Carbon nano-film [24] | 155º | 474 kPa |
| Etched and anodized Al plate with polydimethylsiloxane coating [25] | 159º | 178 kPa |
| Etched and anodized Al plate with fluorination treatment [25] | 161º | 190 kPa |
| Etched Al plate with micro array structure [26] | 155º | 125 kPa |
| Etched Al plate with micro array structure with heating [26] | 152º | 210 kPa |
| Nano-grass-coated microarray-structured Ti6Al4V [27] | 172º | 106 kPa |
| Nanopillar-structured epoxy surface [28] | 154.3º | 7.3 kPa |
| Fluorinated microcone-array Cu plate decorated by CuO microflower and nanograss [29] | 161.2º | 1.7 kPa |
| Fluorinated microcone-array Cu plate decorated by CuO nanograss [29] | / | 26.4 kPa |
| Fluorinated microcone-array Cu plate [29] | / | 208.2 kPa |
| Perfluorodecyltriethoxysilane-grafted etched Al plate [30] | 160.3º | 55.4 kPa |
| Coral-like silicone nanofilament coating [31] | 171º | 10 kPa |
| Poly(vinylidene fluoride-co-hexafluoropropylene) nanofibers on Al alloy plate [32] | 162.0º | 128 kPa |
| ZnO nanorods on Zn-plated steel substrate [33] | 171º | 6 kPa |
| Micropillar of carbon nanotube/polydimethylsiloxane [34] | 165º | 84 kPa |
| Cu(OH)_2_ nanowires on polydimethylsiloxane microcone arrays [35] | 160º | 44.1 kPa |
| Siloxane-modified polyurethane with SiO_2_ nanoparticles [36] | 163.1º | 21.2 kPa |
| Nanostructured woven wire cloths [37] | ⁓160º* | ⁓180 kPa |
| Poly(tetrafluoroethylene)/nanodiamond/MXene composite on silicone film [38] | 160.2º | 56.4 kPa |

*: advancing contact angle

**References for Supporting Information:**

[1] Farhadi S, Farzaneh M, Kulinich SA. Anti-icing performance of superhydrophobic surfaces. Appl Surf Sci. 2011;257(14):6264–6269. doi: 10.1016/j.apsusc.2011.02.057.

[2] Yang S, Xia Q, Zhu L, et al. Research on the icephobic properties of fluoropolymer-based materials. Appl Surf Sci. 2011;257(11):4956–4962. doi: 10.1016/j.apsusc.2011.01.003.

[3] Kulinich SA, Farhadi S, Nose K, et al. Superhydrophobic surfaces: are they really ice-repellent? Langmuir. 2011;27(1):25–29. doi: 10.1021/la104277q.

[4] Chen J, Liu J, He M, et al. Superhydrophobic surfaces cannot reduce ice adhesion. Appl Phys Lett. 2012;101(11):18–21. doi: 10.1063/1.4752436.

[5] Dodiuk H, Kenig S, Dotan A. Do self-cleaning surfaces repel ice? J Adhes Sci Technol. 2012;26(4-5):701–714. doi: 10.1163/016942411X575933.

[6] Wang Y, Xue J, Wang Q, et al. Verification of icephobic/anti-icing properties of a superhydrophobic surface. ACS Appl Mater Interfaces. 2013;5(8):3370–3381. doi: 10.1021/am400429q.

[7] Arianpour F, Farzaneh M, Kulinich SA. Hydrophobic and ice-retarding properties of doped silicone rubber coatings. Appl Surf Sci. 2013;265:546–552. doi: 10.1016/j.apsusc.2012.11.042.

[8] Ge L, Ding G, Wang H, et al. Anti-icing property of superhydrophobic octadecyltrichlorosilane film and its ice adhesion strength. J Nanomater. 2013;2013:278936. doi: 10.1155/2013/278936.

[9] Foroughi Mobarakeh L, Jafari R, Farzaneh M. The ice repellency of plasma polymerized hexamethyldisiloxane coating. Appl Surf Sci. 2013;284:459–463. doi: 10.1016/j.apsusc.2013.07.119.

[10] He Y, Jiang C, Cao X, et al. Reducing ice adhesion by hierarchical micro-nano-pillars. Appl Surf Sci. 2014;305:589–595. doi: 10.1016/j.apsusc.2014.03.139.

[11] Bharathidasan T, Kumar SV, Bobji MS, et al. Effect of wettability and surface roughness on ice-adhesion strength of hydrophilic, hydrophobic and superhydrophobic surfaces. Appl Surf Sci. 2014;314:241–250. doi: 10.1016/j.apsusc.2014.06.101.

[12] Momen G, Jafari R, Farzaneh M. Ice repellency behaviour of superhydrophobic surfaces: effects of atmospheric icing conditions and surface roughness. Appl Surf Sci. 2015;349:211–218. doi: 10.1016/j.apsusc.2015.04.180.

[13] Wang Y, Li M, Lv T, et al. Influence of different chemical modifications on the icephobic properties of superhydrophobic surfaces in a condensate environment. J Mater Chem A. 2015;3(9):4967–4975. doi: 10.1039/c4ta07077a.

[14] Brassard JD, Sarkar DK, Perron J, et al. Nano-micro structured superhydrophobic zinc coating on steel for prevention of corrosion and ice adhesion. J Colloid Interface Sci. 2015;447:240–247. doi: 10.1016/j.jcis.2014.11.076.

[15] Chanda J, Ionov L, Kirillova A, et al. New insight into icing and de-icing properties of hydrophobic and hydrophilic structured surfaces based on core-shell particles. Soft Matter. 2015;11(47):9126–9134. doi: 10.1039/c5sm02143j.

[16] Zheng S, Li C, Fu Q, et al. Fabrication of a micro-nanostructured superhydrophobic aluminum surface with excellent corrosion resistance and anti-icing performance. RSC Adv. 2016;6(83):79389–79400. doi: 10.1039/c6ra13447e.

[17] Ling EJY, Uong V, Renault-Crispo JS, et al. Reducing ice adhesion on nonsmooth metallic surfaces: wettability and topography effects. ACS Appl Mater Interfaces. 2016;8(13):8789–8800. doi: 10.1021/acsami.6b00187.

[18] Wang Y, Liu J, Li M, et al. The icephobicity comparison of polysiloxane modified hydrophobic and superhydrophobic surfaces under condensing environments. Appl Surf Sci. 2016;385:472–480. doi: 10.1016/j.apsusc.2016.05.117.

[19] Wang G, Shen Y, Tao J, et al. Fabrication of a superhydrophobic surface with a hierarchical nanoflake-micropit structure and its anti-icing properties. RSC Adv. 2017;7(16):9981–9988. doi: 10.1039/c6ra28298a.

[20] Nguyen TB, Park S, Lim H. Effects of morphology parameters on anti-icing performance in superhydrophobic surfaces. Appl Surf Sci. 2018;435:585–591. doi: 10.1016/j.apsusc.2017.11.137.

[21] Jin M, Shen Y, Luo X, et al. A combination structure of microblock and nanohair fabricated by chemical etching for excellent water repellency and icephobicity. Appl Surf Sci. 2018;455:883–890. doi: 10.1016/j.apsusc.2018.06.043.

[22] Wu X, Silberschmidt V V., Hu ZT, et al. When superhydrophobic coatings are icephobic: role of surface topology. Surf Coatings Technol. 2019;358:207–214. doi: 10.1016/j.surfcoat.2018.11.039.

[23] Shen Y, Wu Y, Tao J, et al. Spraying fabrication of durable and transparent coatings for anti-icing application: dynamic water repellency, icing delay, and ice adhesion. ACS Appl Mater Interfaces. 2019;11(3):3590–3598. doi: 10.1021/acsami.8b19225.

[24] Xu Y, Zhang G, Li L, et al. Icephobic behaviors of superhydrophobic amorphous carbon nano-films synthesized from a flame process. J Colloid Interface Sci. 2019;552:613–621. doi: 10.1016/j.jcis.2019.05.096.

[25] Barthwal S, Lee B, Lim SH. Fabrication of robust and durable slippery anti-icing coating on textured superhydrophobic aluminum surfaces with infused silicone oil. Appl Surf Sci. 2019;496:143677. doi: 10.1016/j.apsusc.2019.143677.

[26] Li X, Wang G, Moita AS, et al. Fabrication of bio-inspired non-fluorinated superhydrophobic surfaces with anti-icing property and its wettability transformation analysis. Appl Surf Sci. 2020;505:144386. doi: 10.1016/j.apsusc.2019.144386.

[27] Liu R, Chi Z, Cao L, et al. Fabrication of biomimetic superhydrophobic and anti-icing Ti6Al4V alloy surfaces by direct laser interference lithography and hydrothermal treatment. Appl Surf Sci. 2020;534:147576. doi: 10.1016/j.apsusc.2020.147576.

[28] Jia Z, Shen Y, Tao J, et al. Understanding the solid–ice interface mechanism on the hydrophobic nano-pillar structure epoxy surface for reducing ice adhesion. Coatings. 2020;10(11):1043. doi: 10.3390/coatings10111043.

[29] Pan R, Zhang H, Zhong M. Triple-scale superhydrophobic surface with excellent anti-icing and icephobic performance via ultrafast laser hybrid fabrication. ACS Appl Mater Interfaces. 2021;13(1):1743–1753. doi: 10.1021/acsami.0c16259.

[30] Zhang B, Xu W, Xia DH, et al. Comparison study of self-cleaning, anti-icing, and durable corrosion resistance of superhydrophobic and lubricant-infused ultraslippery surfaces. Langmuir. 2021;37(37):11061–11071. doi: 10.1021/acs.langmuir.1c01684.

[31] Bottone D, Donadei V, Niemelä H, et al. Coral-like silicone nanofilament coatings with extremely low ice adhesion. Sci Rep. 2021;11:20427. doi: 10.1038/s41598-021-98215-1.

[32] Vicente A, Rivero PJ, García P, et al. Icephobic and anticorrosion coatings deposited by electrospinning on aluminum alloys for aerospace applications. Polymers (Basel). 2021;13(23):4164. doi: 10.3390/polym13234164.

[33] Balordi M, Pini F, Santucci de Magistris G. Superhydrophobic ice-phobic zinc surfaces. Surf Interfaces. 2022;30:101855. doi: 10.1016/j.surfin.2022.101855.

[34] Sun Y, Wang Y, Liang W, et al. In situ activation of superhydrophobic surfaces with triple icephobicity at low temperatures. ACS Appl Mater Interfaces. 2022;14(43):49352–49361. doi: 10.1021/acsami.2c15075.

[35] Chen C, Tian Z, Luo X, et al. Micro-nano-nanowire triple structure-held pdms superhydrophobic surfaces for robust ultra-long-term icephobic performance. ACS Appl Mater Interfaces. 2022;14(20):23973–23982. doi: 10.1021/acsami.2c02992.

[36] Zhao Y, Hao T, Wu W, et al. A novel moisture-controlled siloxane-modified hyperbranched waterborne polyurethane for durable superhydrophobic coatings. Appl Surf Sci. 2022;587:152446. doi: 10.1016/j.apsusc.2022.152446.

[37] Wood MJ, Brock G, Debray J, et al. Robust anti-icing surfaces based on dual functionality─microstructurally-induced ice shedding with superimposed nanostructurally-enhanced water shedding. ACS Appl Mater Interfaces. 2022;14(41):47310–47321. doi: 10.1021/acsami.2c16972.

[38] Zhao Y, Yan C, Hou T, et al. Multifunctional Ti_3_C_2_T_x_ MXene-based composite coatings with superhydrophobic anti-icing and photothermal deicing properties. ACS Appl Mater Interfaces. 2022;14(22):26077–26087. doi: 10.1021/acsami.2c07087.


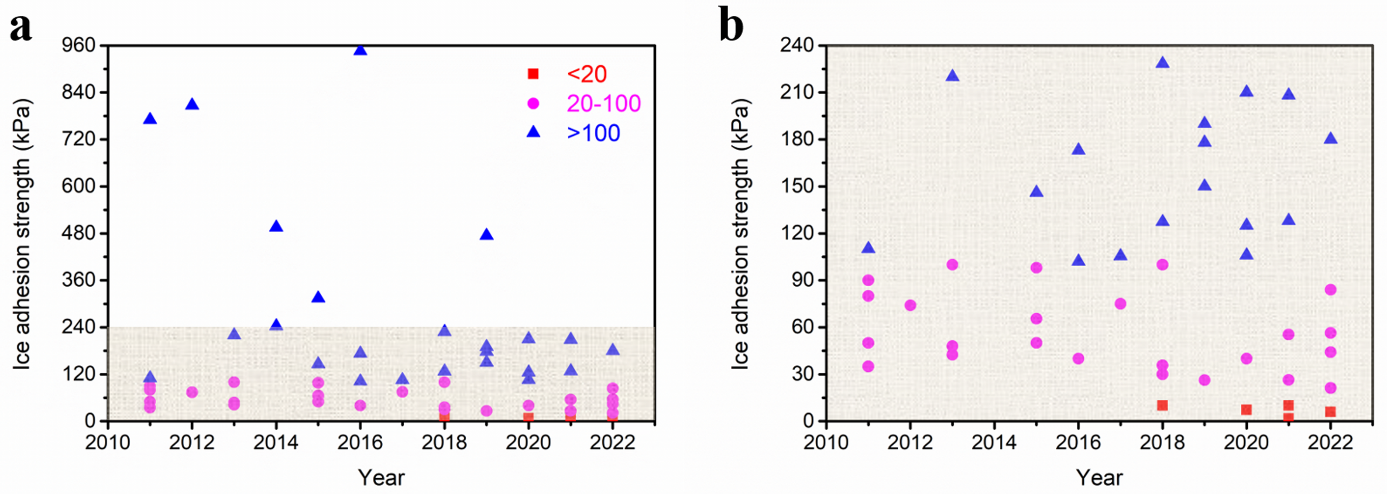


**Figure S1.** IAS data for superhydrophobic surfaces from the literature over the last decade. a) Data between 0 kPa and 960 kPa. b) Data between 0 kPa and 240 kPa.





**Figure S2.** FESEM image of a typical ZnO tetrapod.


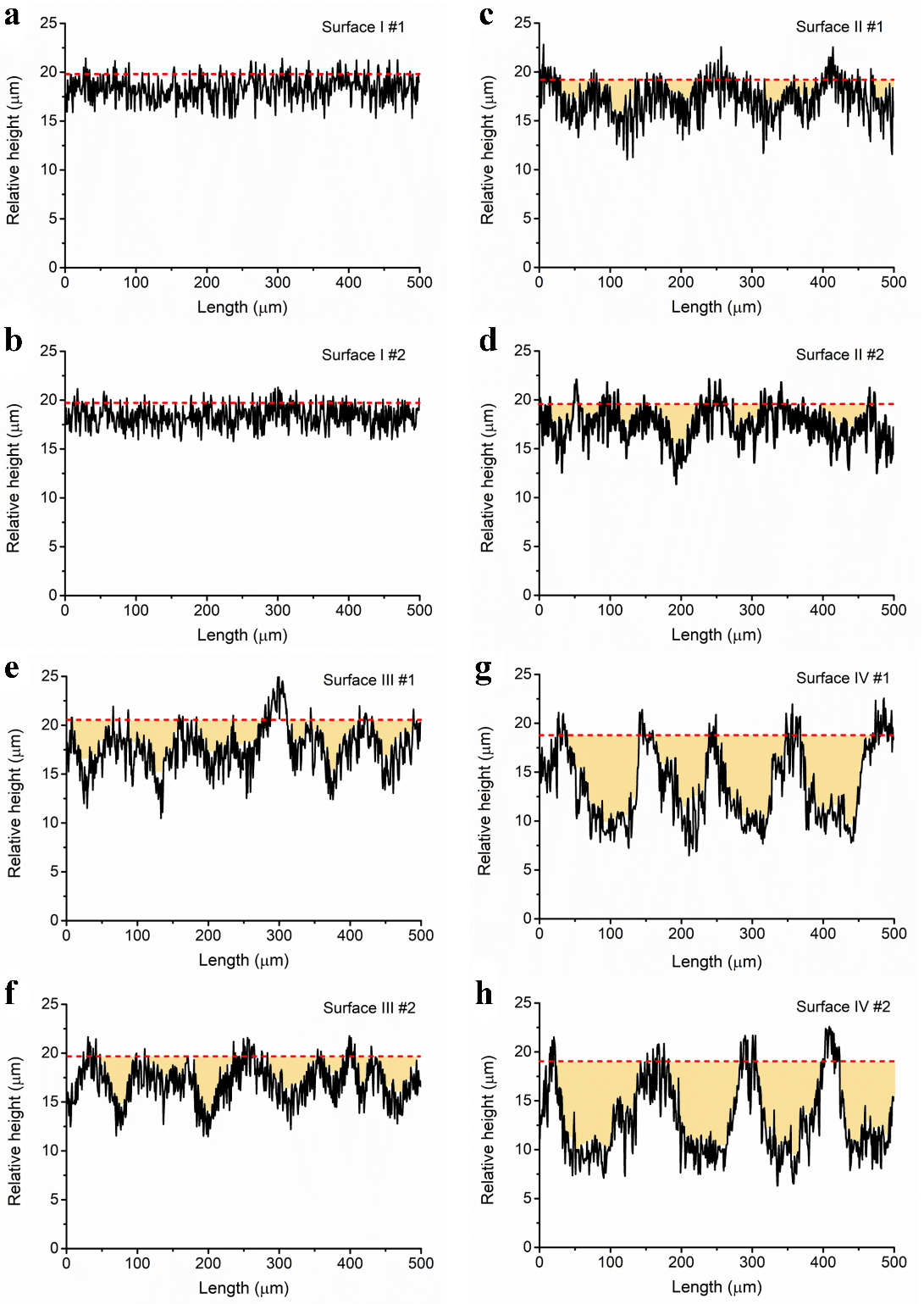


**Figure S3.** a) and b) Surface height profiles of two different zones of surface Ⅰ. c) and d) Surface height profiles of two different zones of surface Ⅱ. e) and f) Surface height profiles of two different zones of surface Ⅲ. g) and h) Surface height profiles of two different zones of surface Ⅳ. The micro-pores are filled with yellow color, and the dotted red lines serve as baselines.


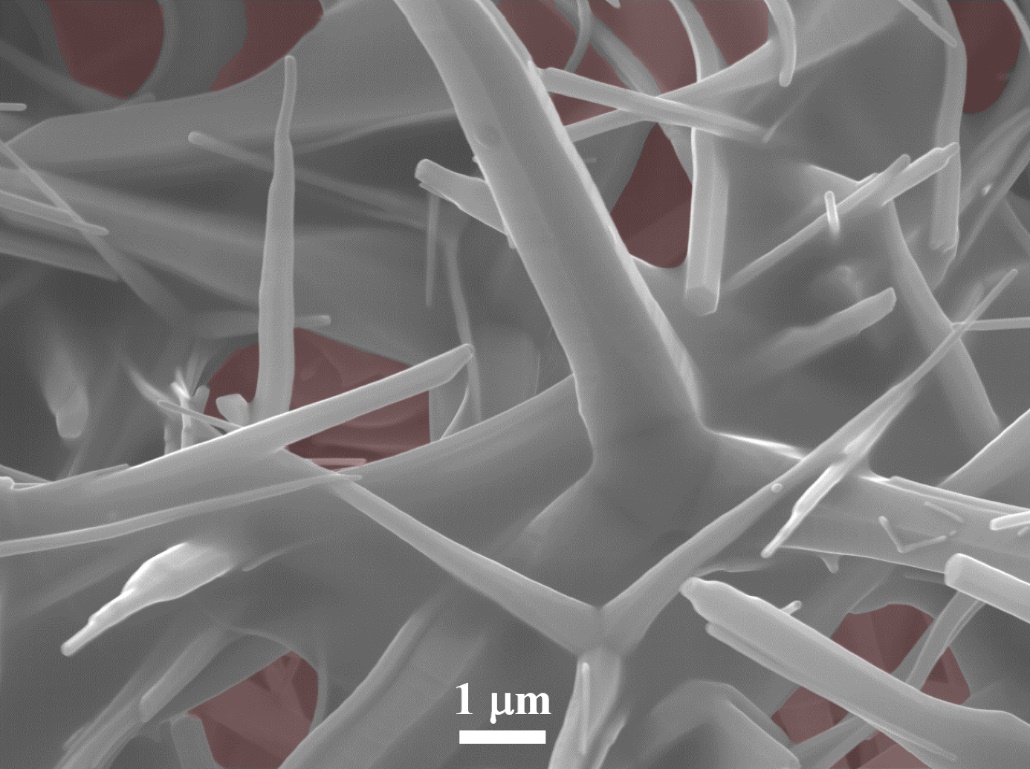


**Figure S4.** High-resolution FESEM image of surface Ⅳ. The pores among the stacked PDMS-coated ZnO tetrapods are filled with red color.


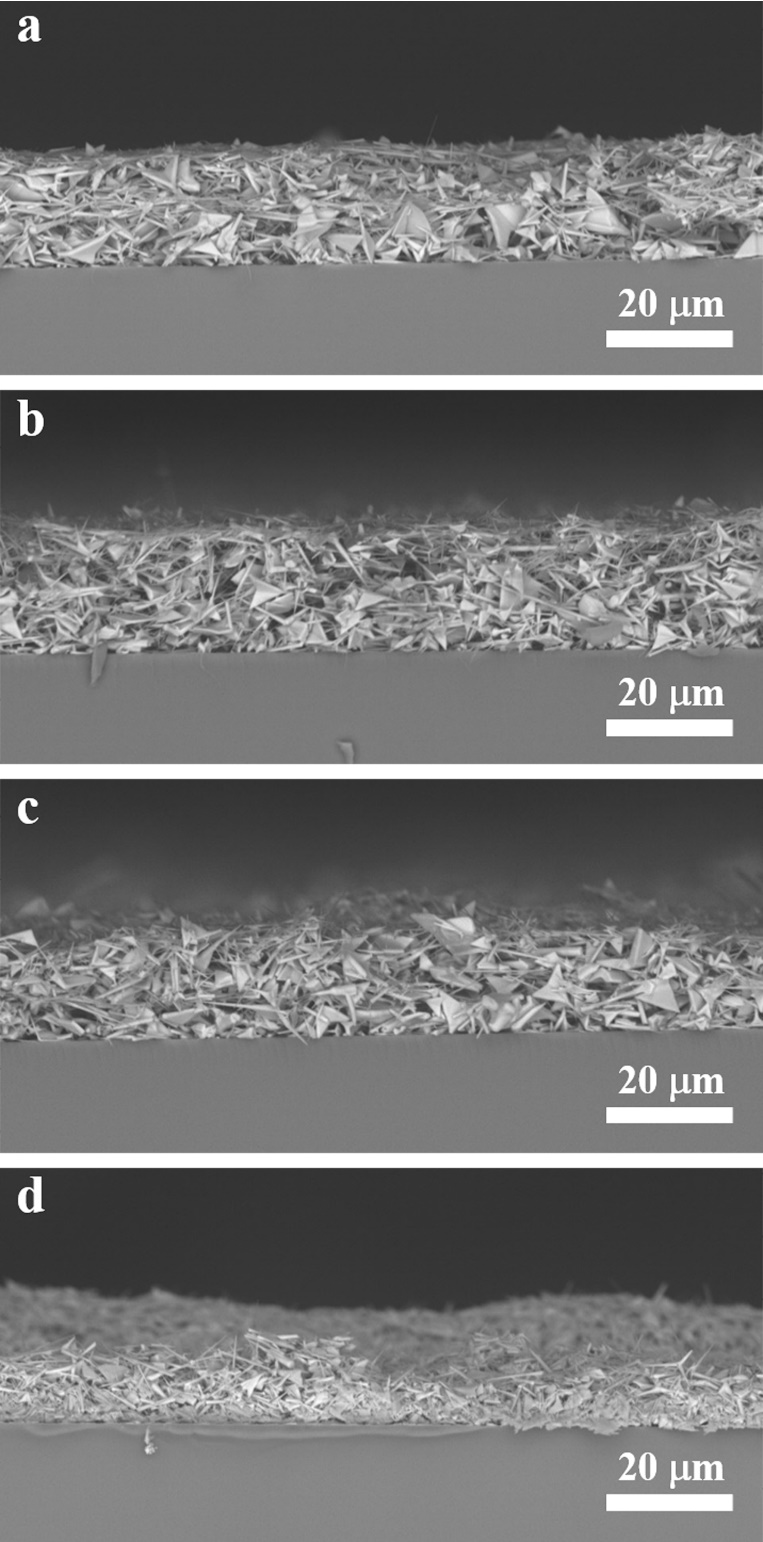


**Figure S5.** Magnified cross-sectional SEM images of a) surface Ⅰ, b) surface Ⅱ, c) surface Ⅲ and d) surface Ⅳ.


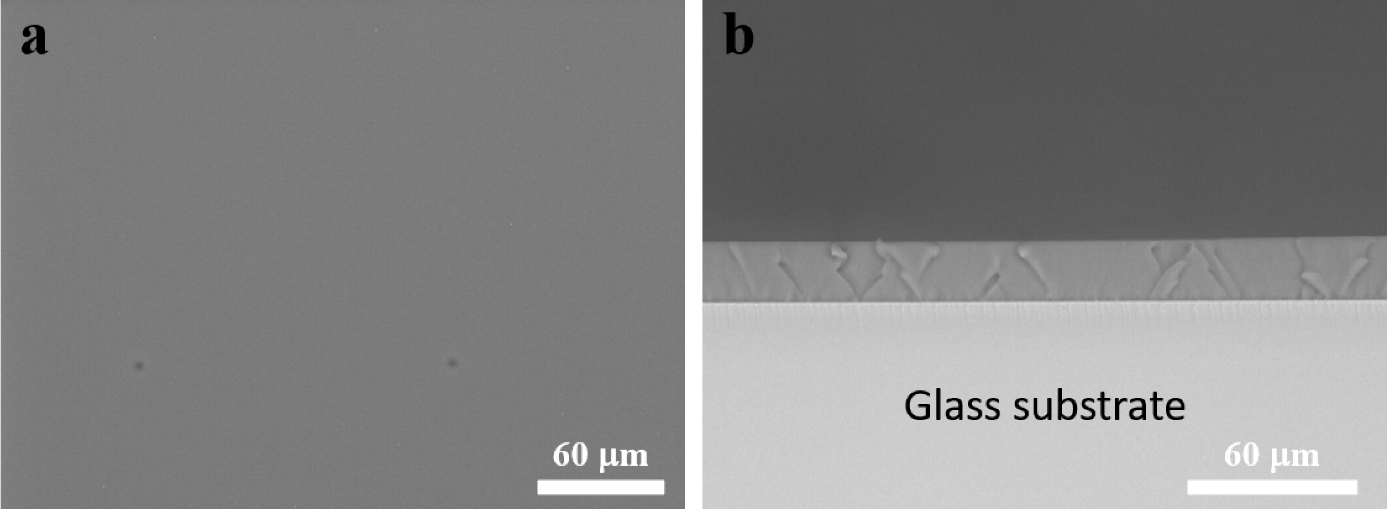


**Figure S6.** a) Top-view SEM image of PDMS surface. b) Cross-sectional SEM image of PDMS surface.


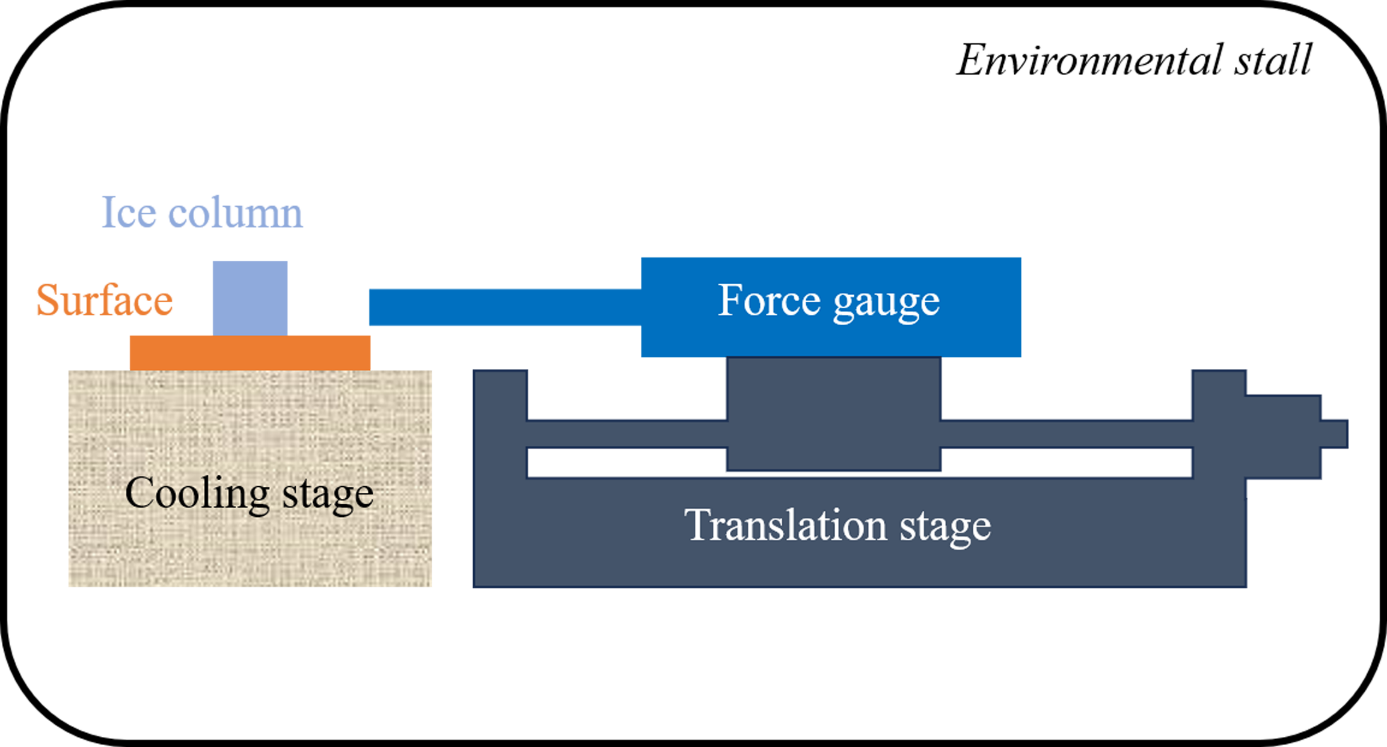


**Figure S7.** Schematics of de-icing test in an environmental stall.





**Figure S8.** Experiemntal dots of IAS vs. roughness factor for four textured surfaces.





**Figure S9.** Experimental dots of IAS vs. arithmetic average roughness (*Ra*) for four textured surfaces.


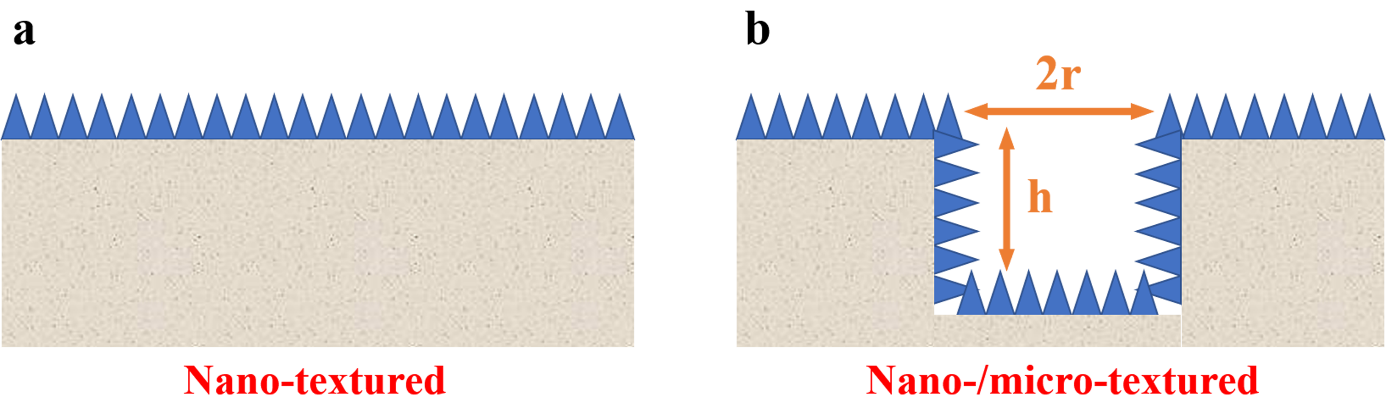


**Figure S10.** Schematics of a) nano-textured surface and b) nano-/micro-textured surface.





**Figure S11.** Experimental dots and linear fitting of IAS vs. areal fraction of micro-textures for four textured surfaces. Two ice types were used.


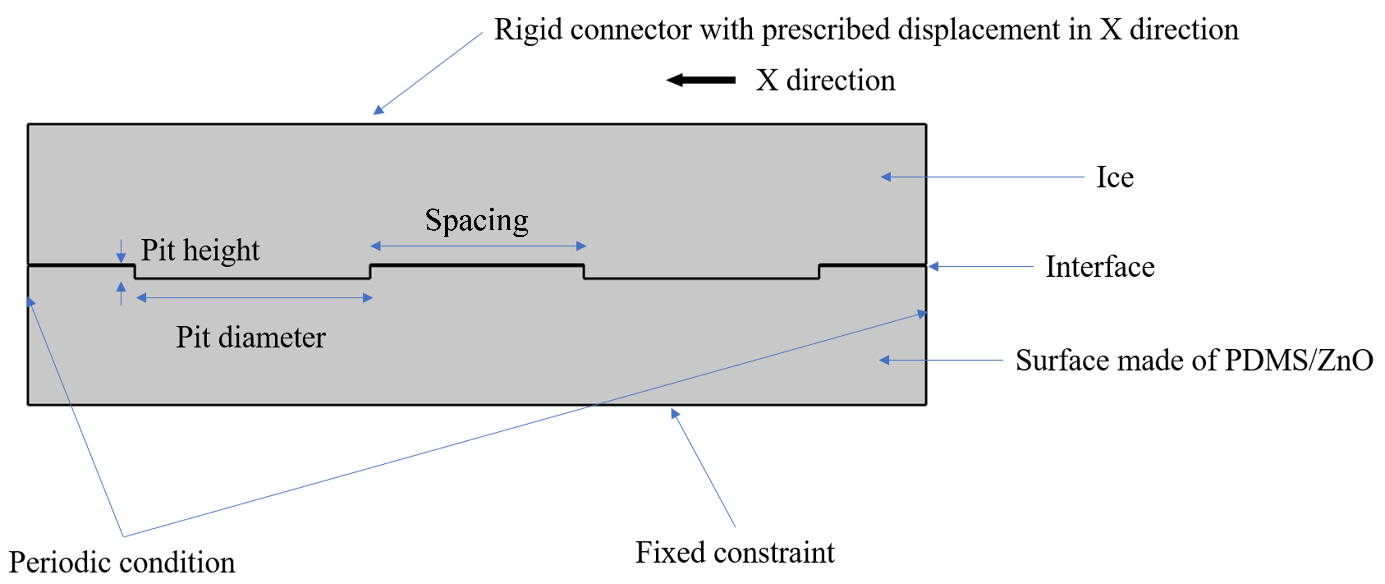


**Figure S12.** Model scheme for ice detachment simulation with geometry and boudary conditions.
